# Evolution of a Large-Scale Virtual Nursing Program: Utilization, Geographic Reach, and Cost Comparison at a Tertiary Care Center in Saudi Arabia, November 2021–July 2025

**DOI:** 10.3390/healthcare14193181

**Published:** 2026-09-25

**Authors:** Khadijah Hadi Maqadi, Waad A. Asiri, Ahmad Aboshaiqah, Ahmad M. Rayani

**Affiliations:** 1 Nursing Practice Department, King Faisal Specialist Hospital and Research Center, P.O. Box 3354, Riyadh 11211, Saudi Arabia; 2 Research and Studies Department, Authority for the Care of Persons with Disabilities (APD), Riyadh 13311, Saudi Arabia; wasiri@apd.gov.sa; 3 Department of Nursing Administration and Education, College of Nursing, King Saud University, Riyadh 11421, Saudi Arabia; aaboshaiqah@ksu.edu.sa; 4 Department of Community and Psychiatric Mental Health Nursing, College of Nursing, King Saud University, Riyadh 11421, Saudi Arabia; arayani@ksu.edu.sa

**Keywords:** virtual nursing, telenursing, telehealth, digital health, healthcare access, healthcare utilization, cost analysis, Saudi Arabia

## Abstract

**Highlights:**

**What are the main findings?**

- The program expanded during the observed period from November 2021 through July 2025, with the recorded number of unique patients increasing from 786 in November–December 2021 to 15,672 in January–July 2025, while the proportion from the Central Region decreased from 99.2% to 60.5%.
- Observed virtual encounters had a 33.1% lower observed mean cost per encounter than physical encounters (625.61 vs. 934.48 Saudi riyals). However, service delivery was overwhelmingly telephone-based (99.9%), and patient-seen rates varied across different clinical groups, though not across years.

**What are the implications of the main findings?**

- The expansion in observed patient volume and geographic distribution describes substantial changes in program activity during the evaluation period, although the study did not directly measure changes in healthcare access or patient travel burden.
- The lower observed mean cost per virtual encounter provides a descriptive basis for further economic evaluation, while the predominance of telephone consultations and variation in operational indicators highlight areas for future assessment of service delivery.

**Abstract:**

**Background/Objectives**: Virtual nursing is a component of digital healthcare delivery, but evidence describing the long-term development, geographic expansion, operational performance, and economic implications is limited. This study evaluated evolution of the Virtual Nursing Program at King Faisal Specialist Hospital & Research Centre (KFSH&RC) from November 2021 to July 2025. **Methods**: A retrospective observational program evaluation was conducted using data from November 2021 to July 2025. Data included patient characteristics, geographic distribution, consultation volume and modality, clinical-group activity, no-show rates, and comparative costs of virtual and physical clinic encounters. Annual unique-patient counts described patient reach, whereas patients seen reflected service utilization and activity volume and could include multiple visits or appointments by the same patient. **Results**: Patients Seen increased from 662 in November–December 2021 to 15,323 in 2022, 32,630 in 2023, and 51,008 in 2024, reaching 38,471 during January–July 2025. The observed calendar-year number of unique patients was 786 during November–December 2021, 9741 in 2022, 6135 in 2023, 13,792 in 2024, and 15,672 during January–July 2025. Geographic reach expanded, with the proportion of patients from the Central Region decreasing from 99.2% to 60.5%. Also, 89,245 consultations were recorded, of which 89,185 (99.9%) were telephone-based. Medical Nursing (30,330), Surgery–Perioperative (21,051), and Cancer Center of Excellence (11,739) had the highest cumulative appointment volumes. In the comparative cost dataset, 46,345 virtual and 331,710 physical encounters were recorded, with mean costs per visit of 625.61 and 934.48 Saudi riyals, respectively, representing a 33.1% lower observed mean cost per virtual encounter. **Conclusions**: We demonstrated expansion in patient reach, geographic coverage, and service diversification during the November 2021–July 2025 evaluation period, while virtual encounters were associated with lower observed costs across all evaluated service lines. Future prospective and comparative studies should evaluate clinical outcomes, patient experience, and full cost-effectiveness.

## 1. Introduction

Telehealth has evolved from a technology-enabled alternative to face-to-face care into an integrated component of contemporary healthcare delivery, with applications ranging from remote consultation and monitoring to patient education and longitudinal follow-up [1]. The expansion of virtual care has been relevant for services that can be delivered without continuous physical examination, including nursing assessment, education, symptom follow-up, chronic disease support, and care coordination [2]. Telenursing, defined as the delivery of nursing practice through telecommunications and information technologies, can include synchronous telephone or video consultations, remote monitoring, asynchronous communication, and mobile-health interventions [3].

The potential value of virtual nursing extends beyond clinical outcomes to healthcare access, service utilization, patient convenience, and health-system efficiency [4]. The choice of virtual modality may influence clinical and service outcomes, with video consultations being more effective than telephone consultations [5]. Telephone-based care nevertheless remains attractive because of its accessibility and low technological requirements, and a systematic review of randomized trials found that telephone consultation can improve the resolution of patient concerns and reduce face-to-face contacts in selected care pathways [6].

These considerations are relevant in Saudi Arabia, where digital transformation has increasingly incorporated telehealth into routine healthcare delivery and where geographic distance between patients and specialized services can be an important barrier to access [7]. A systematic review specifically examining telehealth and nursing care in Saudi Arabia identified evidence that telehealth can strengthen nurse–patient communication and support nursing practice, but emphasized the limited number of nursing-specific studies and the need for additional primary research evaluating telenursing services [7]. Patient-level Saudi studies have also demonstrated favorable perceptions of telehealth [8]. However, Saudi telehealth adoption is influenced by infrastructure, patient and provider attitudes, privacy concerns, digital capabilities, and organizational factors, indicating that successful implementation requires evaluation at health-system and service-delivery levels rather than relying solely on patient acceptability studies [9].

Despite the growing evidence supporting telehealth and telenursing, there are still gaps in understanding how large institutional virtual nursing programs develop and perform over multiple years in real-world healthcare systems [7]. Existing studies have focused on individual diseases, specific interventions, short-term outcomes, or patient attitudes, whereas the systematic review in Saudi Arabia identified only eight relevant studies and specifically highlighted the scarcity of primary research focused predominantly on nursing applications [7]. Similarly, the recent telenursing systematic review in home health care showed heterogeneity in technologies, intervention designs, and outcomes and emphasized that relatively few contemporary studies have evaluated nursing-led virtual care across broader service contexts [10]. Consequently, there is limited evidence describing the longitudinal expansion of a multispecialty virtual nursing program, its ability to extend care geographically, its operational performance across clinical groups, and its economic implications compared with conventional physical care within a single tertiary-care institution. The present study aimed to evaluate the development, implementation, utilization, and expansion of the Virtual Nursing Program at King Faisal Specialist Hospital & Research Centre (KFSH&RC) in Riyadh using routinely collected data from November 2021 to July 2025.

## 2. Materials and Methods

### 2.1. Study Design

This study was a retrospective observational program evaluation of the Virtual Nursing (Tele-Nursing) Program at KFSH&RC, Riyadh, Saudi Arabia. The evaluation examined the development, implementation, expansion, utilization, geographic reach, and operational performance of the program between 1 November 2021 and 31 July 2025.

The study used routinely collected secondary data from institutional virtual nursing databases, electronic health records, scheduling and appointment systems, and other administrative data sources. Because these sources captured different aspects of program activity, separate analytical datasets were retained rather than combining all records into a single denominator. Unique patients were used for demographic and geographic analyses; appointment records were used for appointment volume, clinical-group activity, and appointment-adherence analyses; consultation records were used to characterize consultation modality; clinic-year observations were used for longitudinal operational analyses; and administrative encounter records were used for the virtual-versus-physical cost comparison.

Unique patients represent distinct anonymized patients within the relevant calendar-year dataset, whereas appointments, consultations, and encounters represent service-level records and may include multiple records for the same patient. These measures therefore have different denominators and are not expected to be numerically equivalent (Table S1).

### 2.2. Study Setting and Population

The study was conducted at KFSH&RC in Riyadh, Saudi Arabia, a tertiary/quaternary healthcare institution providing specialized medical and surgical services and incorporating virtual care into routine clinical practice. During the evaluation period, the KFSH&RC Virtual Nursing Program comprised 57 individual virtual nurse clinics distributed across multiple Centers of Excellence, clinical divisions, and nursing specialties. For analytical purposes, these individual clinics were aggregated into 16 major clinical groups according to their principal nursing specialty or organizational affiliation. The number of individual clinics and the number of clinical groups therefore represent different levels of program organization. These services included cardiology, oncology, medical nursing, home health care, obstetrics and gynecology, pediatrics, palliative care, neurosciences, gastroenterology, surgery and perioperative care, urology, genetics, wound and stoma care, tracheostomy/airway care, and patient and family education.

The primary inclusion criterion was at least one documented tele-nursing consultation or virtual nursing appointment between 1 November 2021 and 31 July 2025. Records were excluded from specific analyses when the data required for that analysis were unavailable or insufficient for valid classification. Patients without a documented virtual nursing encounter during the study period were not included.

For analyses of unique patients, patients were identified using the anonymized patient identifier supplied by KFSH&RC. Annual demographic analyses were based on unique patients within each calendar year; therefore, a patient could contribute to more than one annual cohort if the patient received virtual nursing services in multiple years. The 2021 patient-level dataset represents November–December 2021, whereas the 2025 patient-level dataset represents January–July 2025.

### 2.3. Data Sources and Extraction

Data were obtained retrospectively from existing institutional databases and electronic health records. The principal data domains included virtual nursing appointment and encounter records; patient demographic information; patient geographic information; clinic and nursing specialty information; consultation modality; appointment status and no-show information; and administrative and financial data for comparison of virtual and physical care costs, where available. Hospital admission and ED records were reviewed as potentially available outcome data; however, the available extracts did not provide a sufficiently reproducible selection process or validated program-level denominator for inclusion in the final outcome analyses.

Data were extracted by authorized KFSH&RC Health Information Management/Information Technology personnel and provided to the study team in de-identified form. Patient identifiers were replaced by anonymized identifiers before researcher access. Because the available institutional extracts were generated for different operational and analytical purposes, the consultation, appointment, and cost datasets were analyzed separately, with the source, observation period, exclusions, unit of analysis, and denominator specified for each analysis. The analytical dataset was organized at several levels. Patient-level records were used to describe demographic and geographic characteristics; encounter-level data were used to quantify consultation activity and consultation modality; appointment- and encounter-level records were used to quantify service activity and utilization; consultation-level records were used to quantify modality; and clinic-year observations were used to examine changes in operational indicators such as patient volume and no-show rate.

### 2.4. Study Variables and Outcome Measures

Demographic variables included age, sex, and nationality. Age was summarized using the median and interquartile range (IQR) because of the observed non-normal distribution. Sex was categorized as male or female according to the source database. Nationality was categorized as Saudi or non-Saudi. Geographic access was assessed using the patient’s recorded region and city of residence. Regional categories included Central, Northern, Southern, Eastern, and Western Saudi Arabia, with unknown or undefined categories retained where geographic information was unavailable or could not be classified. City-level analyses were conducted using all recorded cities among patients with available and classifiable city information for each year. Annual frequencies and percentages were calculated using the total number of patients with available and classifiable city information in the corresponding year as the denominator. For each demographic and geographic variable, percentages were calculated using the number of patients with non-missing information for that variable as the denominator; the number and proportion of patients with missing or unavailable information were also reported explicitly. The denominator for each percentage is therefore stated in the corresponding table. Annual frequencies and percentages were calculated for each geographic region. City-level analyses identified the most frequently represented cities in each year to characterize the evolution of the program’s geographic reach.

Virtual nursing utilization was assessed using the number of unique patients and the number of virtual nursing consultations/appointments. Unique patients represented distinct patients within each calendar year, whereas patients seen represented service activity and could include repeated visits by the same patient. Monthly and annual patient volumes were summarized to describe changes in program activity over time. Cumulative appointment volume was also calculated for each of the 16 major clinical groups and, where applicable, for individual virtual clinics. Each recorded virtual nursing encounter was classified according to the consultation modality documented in the source database, principally telephone or video. Annual frequencies and percentages of each modality were calculated to characterize the evolution of virtual nursing delivery.

Appointment adherence was evaluated using the no-show rate reported by the virtual nursing program database. At the appointment level, the patient-seen rate was defined at the appointment level as the proportion of eligible scheduled appointments recorded as patient-seen, with the numerator representing appointments classified as seen and the denominator representing eligible scheduled appointments after exclusion of canceled appointments. The no-show rate was defined as the proportion of eligible scheduled appointments classified as no-shows using the same denominator. Cancellations were excluded from both measures because they do not represent an attendance failure by the patient.

Clinic-year observations constituted the analytical level for summaries of patient-seen and no-show rates. These clinic-year rates were subsequently summarized within the 16 clinical groups using medians and interquartile ranges. Calendar years before a clinic became operational were treated as not applicable and were not coded as zero. A zero value was retained only when an operational clinic had no recorded activity or events during an observed clinic-year.

No-show rates were summarized at the clinical-group level using the median and IQR across available clinic-year observations. For longitudinal analyses, clinic-years in which a clinic was not operational were treated as not applicable and were not coded as zero. By contrast, zero values were retained when an active clinic had no recorded activity or events during an observed clinic-year. This approach distinguished structural zeros resulting from non-operation from true zero activity among operational clinics.

For each of the 16 clinical groups, longitudinal changes in service activity were assessed by comparing the first year in which activity was recorded (baseline year) with 2025, using clinic-level activity records for which the baseline and 2025 observations were considered comparable under the prespecified analytical criteria. The baseline/end-point dataset therefore represents a restricted longitudinal analytical subset and is not expected to reproduce the full appointment totals. Absolute change was calculated as activity in 2025 − activity in baseline year. Percentage change was calculated as [(activity in 2025 − activity in baseline year)/activity in baseline year] × 100.

### 2.5. Statistical Analysis

All analyses were performed using SPSS software version 24. Continuous variables were summarized using the mean and standard deviation when appropriate and the median and IQR when distributions were skewed. Categorical variables were summarized using frequencies and percentages.

Because clinic-level observations were repeated across calendar years and the number of observations contributed by individual clinics was unequal, longitudinal operational measures were primarily summarized descriptively at the clinic-year level. Patient-seen and no-show rates were summarized using medians and interquartile ranges across available clinic-year observations within each clinical group and calendar year. Longitudinal changes in no-show rates and associations between appointment volume and no-show rate were treated as exploratory analyses because repeated clinic-year observations were not statistically independent and the number of observations was limited and unbalanced across groups. Simple linear regression, Spearman’s rank correlation, and Kendall’s tau were therefore interpreted as exploratory, hypothesis-generating analyses rather than confirmatory tests. No multiplicity adjustment was applied across the separate group-specific exploratory analyses. The number of observations contributing to each analysis was reported explicitly, and clinic-years before clinic activation were treated as not applicable rather than as zero observations.

The distribution of patient-seen rates was compared across the 16 clinical groups using the Kruskal–Wallis test. A separate Kruskal–Wallis test was used to assess differences in patient-seen rates across calendar years. When the overall group comparison was statistically significant, pairwise comparisons were explored using Dunn’s post hoc test with Bonferroni adjustment for multiple comparisons. A two-sided *p*-value < 0.05 was considered statistically significant.

Changes in no-show rates over the study period were examined using simple linear regression, with calendar year treated as the independent variable and the annual/group-specific no-show rate as the dependent variable. The relationship between appointment volume and no-show rate was evaluated using Spearman rank correlation and Kendall’s tau. Because the number of available clinic-year observations varied across clinical groups and was as low as two observations for some longitudinal analyses, these regression and correlation analyses were considered exploratory and hypothesis-generating. No multiplicity adjustment was applied across the separate group-specific regression or correlation analyses; therefore, statistically significant findings from these analyses were interpreted cautiously and were not considered confirmatory.

The analysis required linkage between the patient’s index discharge date and a subsequent inpatient admission date. Where the available data did not provide sufficient temporal information for reliable classification, the record was not considered independently validated for the outcome. For the correlation analyses, *n* represents the number of available clinic-year observations contributing to the analysis after exclusion of observations without the required volume or no-show information.

Where comparable administrative cost data were available, the cost of virtual nursing encounters was compared with the cost of physical clinic encounters across corresponding service lines. The cost dataset covered the available comparable encounter records during the study period (November 2021 through July 2025) and was obtained from the KFSH&RC institutional administrative and financial records. Costs were reported in Saudi riyals as recorded in the institutional dataset and were not inflation-adjusted because the analysis was intended to compare the recorded costs of virtual and physical encounters within the available institutional costing system rather than to estimate costs in a common constant-price year.

The analysis used the encounter-level cost values available in the institutional financial dataset. These recorded costs represent the costs captured and allocated by the hospital’s administrative costing system for the corresponding encounters; the study team did not independently reallocate individual staffing, facility, or overhead costs. Separate patient-incurred costs, including travel and other out-of-pocket expenses, were not included. Similarly, technology procurement, program implementation, and start-up costs were not separately incorporated into the encounter-level comparison because they were not identifiable as distinct components of the available encounter-level cost records. Consequently, the reported values should be interpreted as recorded institutional encounter costs rather than comprehensive societal or program-level costs.

The principal economic measure was cost per encounter, with aggregate costs and encounter volumes also reported. The difference between virtual and physical cost per encounter was calculated both as an absolute difference and as a percentage reduction relative to physical care. Because the virtual and physical encounters were not matched or adjusted for differences in patient characteristics, clinical characteristics, case complexity, utilization patterns, or other potential confounding factors, the comparison was descriptive and was not intended to estimate causal cost savings, incremental costs, or cost-effectiveness.

The Virtual Nursing Program was organized into 16 clinical groups for analyses of program activity, whereas the administrative cost dataset classified encounters into 11 discharge service lines. These two classification systems served different purposes and were not assumed to have a one-to-one correspondence. The cost analysis was therefore conducted using the 11 discharge service lines available in the financial dataset, rather than the 16 virtual nursing clinical groups.

## 3. Results

### 3.1. Overall Evolution of the Virtual Nursing Program

Between November 2021 and July 2025, the Virtual Nursing Program at King Faisal Specialist Hospital and Research Centre expanded from a relatively small, predominantly telephone-based service into a multidisciplinary program spanning 16 major virtual nursing groups and multiple specialty-specific clinics. The corresponding total patients seen were 662 in Nov–Dec 2021, 15,323 in 2022, 32,630 in 2023, 51,008 in 2024, and 38,471 in Jan–Jul 2025, representing service activity rather than unique patients and therefore allowing repeated visits or appointments by the same patient. The observed number of unique patients by calendar year was 786 during November–December 2021, 9741 in 2022, 6135 in 2023, 13,792 in 2024, and 15,672 during January–July 2025. Median patient age increased from 37 years in 2021–2022 to 40 years in 2024–2025, while the age distribution became broader over time, with the IQR increasing from 14 years in 2021 to 29 years in 2024 and 2025 (Table 1).

### 3.2. Geographic Expansion and National Reach

The geographic reach expanded considerably in 2023, when the proportion of patients from the Central Region declined to 71.7%. Patients from the Northern Region accounted for 9.1%, Southern Region 8.4%, Eastern Region 5.9%, and Western Region 4.4%. By 2024 and 2025, only approximately 60% of patients were from the Central Region, while approximately 39% were from other identified regions in each year. In 2025, 12.6% of patients were from the Southern Region, 11.3% from the Northern Region, 8.7% from the Eastern Region, and 6.3% from the Western Region (Table 2).

A similar pattern was observed at the city level. Among patients with available and classifiable city information, Riyadh accounted for 99.8% of patients in November–December 2021 (608/609) and 97.3% in 2022, before declining to 80.0% in 2023 and approximately 70% in 2024 and 2025. By 2025, Buraydah, Hail, Abha, Jazan, Najran, Dammam, Medina, Alahsa, and Sakaka were among the most frequently represented non-Riyadh cities (Table S2).

### 3.3. Monthly Virtual Nursing Utilization

The available monthly activity series covered November 2021 through July 2025. The series showed 45 patients in November 2021 and 617 in December 2021. Monthly activity subsequently increased throughout 2022 and 2023, reaching 2486 patients in December 2022 and 3392 patients in January 2023. During 2024, several months exceeded 4500 patients, including May (4538), July (4801), August (4812), September (4898), October (5899), November (5050), and December (5678). In the available 2025 series, monthly activity reached 7149 patients in July, representing the highest monthly value recorded during the available study-period extract (Table S3).

### 3.4. Consultation Modality

Telephone consultation was the dominant modality throughout the evaluation period. Overall, 89,245 consultations were identified in the consultation-type extract during 2021–2025, of which 89,185 (99.9%) were telephone-based, and 60 (0.1%) were video-based. In 2021 and 2022, all reported consultations were conducted by telephone. In 2023, 17,784 consultations were conducted by telephone and only 2 by video. In 2024, 31,974 telephone consultations and 36 video consultations were recorded. In 2025, 24,186 consultations were conducted by telephone and 22 by video.

### 3.5. Distribution and Expansion of Virtual Nursing Services

The virtual nursing program included 16 major clinical groups during 2021–2025, with variation in activity and timing of implementation. The largest cumulative appointment volumes in the appointment extracts were in Medical Nursing (30,330 appointments), Surgery–Perioperative (21,051), Cancer Center of Excellence (11,739), Obstetrics and Gynecology (9837), Home Health Care (7107), and Gastroenterology (6842). Medical Nursing accounted for activity throughout the entire evaluation period, whereas several specialist groups became operational only after program expansion. For example, Cardiology activity appeared in 2023 and increased to 766 appointments by 2025. Neurosciences increased from 20 appointments in 2022 to 533 in 2025, and Palliative Care increased from 594 appointments in 2024 to 1338 in 2025. At the clinic level, the Cardio Adult VAD Nurse Virtual Clinic increased from 81 appointments in 2024 to 415 in 2025 (+412.3%), the Pre-EMU Admission Virtual Nurse Clinic increased from 17 in 2022 to 476 in 2025 (+2700%), and the Palliative Care Nurse Virtual Clinic increased from 483 in 2024 to 1211 in 2025 (+150.7%). Conversely, some individual clinics experienced complete cessation of recorded activity during the available observation period, including several colorectal, gastroenterology, obstetric/gynecologic, and perioperative clinics (Table S4).

No-show rates varied substantially among clinical groups. The highest median no-show rate was observed in Tracheostomy–Airway Care (23.1%), followed by the Cancer Center of Excellence (15.4%) and Wound–Stoma Care (13.5%). In contrast, the lowest median rates were observed in Home Health Care (1.3%), Obstetrics and Gynecology (1.7%), Palliative Care (2.1%), and Surgery–Perioperative (2.5%) (Table S5).

### 3.6. Longitudinal Changes in Virtual Nursing Activity

Using the restricted clinic-level dataset with comparable baseline and 2025 observations, Medical Nursing increased from 868 appointments in 2021 to 3563 in the underlying baseline/end-point analysis, representing a 310.5% increase. Neurosciences increased by 2700%, Cardiology by 11,500%, Pediatrics by 1503.2%, and Palliative Care by 125.1%. Home Health Care also demonstrated a large relative increase, although its percentage change was driven by a very small baseline volume. In contrast, several clinical groups had no recorded activity in 2025 despite previous activity, including Obstetrics and Gynecology, Wound–Stoma Care, Gastroenterology, and Tracheostomy–Airway Care (Table S6).

### 3.7. Clinic-Level Program Evolution

In the Cancer Center of Excellence, some services expanded, including the Breast Care Education Nurse Virtual Clinic (+573.9%) and Medical Oncology Education Virtual Nurse Clinic (+321.2%), while several colorectal and stoma-related virtual clinics recorded no activity by their last observed year. Within Medical Nursing, the Family Medicine Virtual Nurse Clinic increased from 868 encounters in 2021 to 1320 in 2025 (+52.1%). Several newly introduced pulmonary and specialty services also expanded rapidly, including the Pulmo Intervention Clinical Specialist Nurse Virtual Clinic (+728.6%).

In Home Health Care, the HHC Urgent Virtual Clinic increased from one encounter in 2021 to 112 in 2025, while the HHC Nurse Virtual and HHC Nurse Virtual Walk-In services showed reductions. The Cardio Adult VAD Nurse Virtual Clinic increased from 81 encounters in 2024 to 415 in 2025 (+412.3%), while the Cardiology Nurse Practitioner Virtual Clinic declined by 22.6%.

### 3.8. Differences in Patient-Seen Rates Across Clinical Groups and Years

Patient-seen rate was defined as the proportion of eligible scheduled appointments recorded as patient-seen after exclusion of cancellations. Patient-seen rates varied across the 16 clinical groups in the available clinic-year observations. Because clinic-year observations were repeated within clinics and were not statistically independent, differences between groups and years are presented descriptively rather than interpreted as confirmatory inferential comparisons (Table S5).

### 3.9. No-Show Rate Trends and Associations

The longitudinal analyses of no-show rates were exploratory because the available clinic-year observations were repeated within clinics, unequal across clinical groups, and limited for several groups. A downward pattern was observed in the Cancer Center of Excellence, while Genetics showed an upward pattern; most other groups did not show clear temporal patterns (Table S7). Because these analyses were exploratory and affected by repeated observations and multiple comparisons, the observed group-specific coefficients and *p*-values should not be interpreted as confirmatory evidence of temporal change.

Appointment volume showed little overall association with no-show rate in the available observations. Spearman correlation analysis yielded r = 0.062 (*p* = 0.472), while Kendall’s tau was 0.033 (*p* = 0.566) across 138 clinic-year observations. At the subgroup level, correlations were observed for the Cancer Center of Excellence and Home Health Care; however, these findings should be considered hypothesis-generating given the repeated clinic-year structure, limited observations in some groups, and absence of multiplicity adjustment (Table S8).

### 3.10. Cost Comparison

A total of 378,055 encounters were recorded across the included discharge service lines, comprising 331,710 physical clinic encounters and 46,345 virtual clinic encounters. Virtual clinics accounted for approximately 12.3% of all encounters during the study period. The total cost associated with physical clinic encounters was 309,975,929, compared with 28,993,908 for virtual clinic encounters. The overall mean cost per visit was 934.48 Saudi riyals for physical clinics and 625.61 Saudi riyals for virtual clinics, representing a 33.1% lower observed mean cost per virtual encounter.

At the service-line level, virtual clinics had a lower cost per visit than physical clinics across all 11 service lines. The greatest relative reduction was observed in the Neuroscience Centre–Admin, where the cost per virtual visit was 332 Saudi riyals compared with 715 Saudi riyals for a physical visit, corresponding to a 53.6% lower observed mean cost per virtual encounter. This was followed by Obstetrics and Gynecology (415 vs. 554 Saudi riyals; 25.1% lower observed mean cost), Dermatology (499 vs. 725 Saudi riyals; 31.2% lower observed mean cost), and Family Medicine/Polyclinics (580 vs. 605 Saudi riyals; 4.1% lower observed mean cost). Among the higher-volume service lines, Family Medicine/Polyclinics had the largest number of virtual encounters (19,917), followed by Medicine (11,513) and Obstetrics and Gynecology (5657). The cost per virtual visit was 721 Saudi riyals in medicine compared with 1105 Saudi riyals for physical visits, a 34.8% lower observed mean cost. Similarly, in Oncology Centre, the cost per visit decreased from 1034 Saudi riyals for physical care to 857 Saudi riyals for virtual care, a 17.1% lower observed mean cost. The largest absolute difference in recorded mean cost per encounter was observed in Orthopedic Surgery, where virtual and physical encounters had recorded mean costs of 1380 and 1945 Saudi riyals, respectively, an absolute difference of 565 Saudi riyals per encounter (29.1% lower observed mean cost) (Table 3).

## 4. Discussion

The trajectory of the Tele-Nursing Program at KFSH&RC reflects a remarkable evolution from concept to institutional integration. This initiative was originally conceived as a strategic improvement and business case to enhance nursing service delivery, particularly in outpatient settings. The onset of the COVID-19 pandemic in early 2020 catalyzed its activation, transforming a visionary idea into an urgent operational necessity. During the peak of the COVID-19 pandemic, the Tele-Nursing Program was launched as a pilot initiative to ensure continuity of care amidst lockdowns and quarantine restrictions. Initially, patient contact was conducted exclusively via telephone, allowing nurses to assess patients’ conditions, schedule follow-up appointments, and coordinate with physicians for medication prescriptions or clinical clarifications. This phone-based model provided a rapid, low-barrier solution to maintain essential nursing services during a time of crisis. Following the initial success and positive patient feedback, the program transitioned to a more structured virtual platform integrated within the hospital’s digital infrastructure. Secure video consultations and electronic documentation systems were subsequently introduced as part of program development. To support this transition, structured educational sessions and unit-based rounds were conducted to introduce the concept to nursing teams, clarify its benefits, and assess feasibility across outpatient units. Head nurses and frontline staff were actively engaged in co-designing workflows, receiving tailored training, and being equipped with the necessary digital tools. Collaboration with Health Information Technology specialists supported the integration of virtual platforms and data systems. These implementation activities formed part of the development of the virtual nursing service, although the present study did not formally evaluate sustainability, scalability, implementation effectiveness, or system-level outcomes.

The observed trajectory during November 2021–July 2025 showed that the KFSH&RC Virtual Nursing Program evolved from a relatively small service into a large, multispecialty model of care with greater patient reach, broader geographic coverage, and lower observed cost per encounter than corresponding physical clinic care. The number of unique patients recorded in the calendar-year datasets increased from 786 during November–December 2021 to 15,672 during January–July 2025; however, these figures should not be interpreted as a standardized annual growth measure because the available observation periods were incomplete and differed across the study period. The magnitude of this longitudinal institutional expansion is notable because the recent literature generally reports telehealth growth at the service or population level, but relatively few studies describe the evolution of a nursing-led virtual-care program over the study period within a single tertiary health system. A scoping review of nurse practitioner-led telehealth concluded that telehealth improved access across multiple service domains but also emphasized that the evidence base remained heterogeneous with limited longitudinal implementation evidence, which makes the present institutional evaluation particularly relevant [11].

The progressive geographic diversification observed in this study, with the proportion of patients from outside the Central Region increasing over the evaluation period, documents a broader geographic distribution of recorded virtual nursing patients. However, the present study did not directly measure healthcare access, travel distance, travel costs, unmet need, or inequities in access. The observed geographic distribution should therefore not be interpreted as direct evidence of improved access or reduced geographic barriers. This finding is consistent with a systematic review of strategies to improve rural primary healthcare access, which identified telemedicine among the major approaches capable of extending care to geographically underserved communities [12]. However, the present study did not directly measure healthcare access, travel distance, travel costs, unmet need, or changes in access among previously underserved populations. Therefore, the observed geographic distribution should not be interpreted as direct evidence of improved access or reduced geographic barriers. In Saudi Arabia, this interpretation is particularly relevant because a cross-sectional study specifically examining telemedicine access among patients with chronic conditions identified disparities in telemedicine use, demonstrating that access is influenced by socioeconomic and demographic characteristics rather than geography alone [13]. The expansion observed at KFSH&RC may therefore show the ability of a well-established tertiary virtual-care infrastructure to overcome travel requirements, particularly for patients requiring repeated specialist nursing follow-up, although the present study cannot determine whether utilization increased preferentially among previously underserved populations. This distinction is important because telehealth does not eliminate inequity. A large-scale analysis found that rural and low-income adults were less likely to report telehealth access than their comparison groups, despite willingness to use telehealth [14].

The reduction in Riyadh’s share of the virtual nursing population from 99% in 2021 to approximately 70% in 2024–2025 documents a shift in the geographic distribution of recorded virtual nursing patients toward a greater representation from outside Riyadh. This pattern is compatible with Saudi evidence showing that telemedicine has become acceptable across different regions and population groups, including a Saudi study of 641 patients in primary healthcare settings in which 82.7% reported satisfaction with telemedicine and 84.1% expressed a positive attitude toward it [8]. Similarly, another Saudi study involving 720 patients attending outpatient telemedicine clinics reported acceptance of telemedicine across patients from different regions, suggesting that virtual models can be acceptable beyond the population surrounding the service provider [15]. The present data cannot determine whether the observed geographic diversification resulted from increased institutional capacity, changes in referral patterns, changes in patient preferences, or other factors [16].

We found the predominance of telephone-based virtual nursing, with approximately 99.9% of recorded consultations delivered by telephone and only 0.1% by video. This pattern differs from several telemedicine studies in which video became the dominant modality, but it is consistent with evidence that telephone care remains an effective and pragmatic modality when clinical assessment does not require visual examination or high-bandwidth interaction. A systematic review comparing telephone and video telehealth found that video was superior or equivalent to telephone for most clinical outcomes, but telephone remained clinically effective in a subset of studies and the two modalities were frequently equivalent [17]. Another systematic review of 79 comparative studies similarly found video to be the most effective modality in 50% of studies compared with only 4% favoring telephone, while 35% found the two modalities equivalent, suggesting that modality selection should be driven by clinical requirements rather than an assumption that video is always preferable [5]. The high use of telephone in our program documents the operational predominance of a low-bandwidth modality within the evaluated service. The available data do not allow assessment of whether this pattern was related to internet access, digital literacy, patient preference, clinical suitability, or other organizational factors. Because 99.9% of recorded consultations were telephone-based, the findings may have limited generalizability to virtual nursing programs in which video consultation, remote monitoring, asynchronous communication, or other digital modalities constitute a larger proportion of service delivery.

The diversification of the program across 16 clinical groups, together with simultaneous expansion, reduction, and discontinuation of individual clinics, documents variation in service activity and timing of implementation across clinical groups. The available data do not establish whether these changes represented service optimization, changes in clinical demand, organizational restructuring, or other operational factors. The prominence of Medical Nursing, Surgery–Perioperative, Cancer Center of Excellence, Obstetrics and Gynecology, Home Health Care, and Gastroenterology is consistent with the literature showing that tele-nursing has been most extensively used for chronic disease management, symptom monitoring, education, and follow-up rather than for activities requiring direct physical examination. A systematic review and meta-analysis of 17 telenursing studies in home health care found that 70% of targeted conditions were chronic obstructive pulmonary disease or heart failure and that telemonitoring and synchronous telephone/video nursing were the most common intervention types [10]. The same review found significant reductions in hospital admissions (standardized mean difference [SMD]: −0.18, 95% CI: −0.43 to −0.02) and ED visits (SMD: −0.28, 95% CI: −0.45 to −0.10), supporting the clinical plausibility of using nursing-led virtual services for longitudinal follow-up and symptom surveillance [10]. The more heterogeneous service pattern in our study can be explained by the institutional nature of KFSH&RC, where specialty-specific virtual clinics can be created, modified, merged, or discontinued in response to local clinical demand rather than following a single standardized intervention protocol.

The variation in no-show rates across clinical groups was another important finding, ranging from a median of 1.3% in Home Health Care to 23.1% in Tracheostomy–Airway Care, with the Cancer Center of Excellence also showing a relatively high median rate of 15.4%. These values are compatible with published evidence indicating that virtual modalities can reduce missed appointments, although the magnitude of benefit varies according to patient population and modality. In the study by Muppavarapu et al. on 13,916 pre-pandemic and 13,251 pandemic-period scheduled psychiatric visits, the overall no-show rate decreased from 18.1% to 15.3% after the transition toward telehealth, and telephone visits had lower no-show rates than face-to-face visits [18]. Similarly, a retrospective cohort study involving 3,105,382 scheduled visits found that telephone and video visits were associated with 50% and 15% lower adjusted odds of no-show, respectively, compared with in-person care [19]. The lack of a significant correlation between appointment volume and no-show rate in our study, however, suggests that clinic volume alone does not explain adherence behavior, and the significant positive correlations observed in the Cancer Center and Home Health Care may instead show differences in case complexity, appointment scheduling patterns, follow-up intensity, or patient population. This interpretation is consistent with evidence that no-show behavior is affected by several patient- and system-level factors and cannot be attributed solely to the mode of consultation [19].

The significant between-group difference in patient-seen rates in our study, together with the absence of a significant difference across years, suggests that operational activity was driven more strongly by specialty-specific service design than by calendar-year effects. However, because the patient-seen analyses were based on available clinic-level observations and clinical groups contributed unequal numbers of observations, these findings should be interpreted cautiously. A meta-analysis identified different technology and nursing intervention types and reported heterogeneous effects across healthcare utilization, physiological outcomes, psychological outcomes, and quality of life [10]. The absence of significant post hoc pairwise differences after Bonferroni adjustment in our study also indicates that the overall group-level heterogeneity should not be interpreted as evidence that any specific pair of specialties was definitively different, particularly given the unequal and sometimes small numbers of observations contributed by individual groups.

The longitudinal no-show analysis showed a statistically significant decline in the Cancer Center of Excellence but a significant increase in Genetics, whereas most other groups did not demonstrate significant trends. These analyses were exploratory because individual clinical groups contributed only a small number of available clinic-year observations, and no multiplicity adjustment was applied across the group-specific tests. The statistically significant findings should therefore be regarded as hypothesis-generating rather than definitive evidence of temporal change. The mixed pattern contrasts with studies reporting more uniform reductions in missed appointments after telehealth adoption, such as the large cohort in which telephone and video care were associated with lower odds of no-show than in-person care [19]. The discrepancy probably shows the fact that our analysis evaluated longitudinal performance within a virtual system rather than comparing telehealth with physical care, meaning that changes in patient mix, clinic maturity, appointment scheduling, service redesign, and specialty-specific workflow may be more important than consultation modality itself.

The cost analysis showed a lower observed mean cost per encounter for virtual care across each of the 11 evaluated discharge service lines, with an overall recorded mean cost of 625.61 Saudi riyals per virtual encounter compared with 934.48 Saudi riyals per physical encounter, corresponding to a 33.1% lower observed mean cost. Because the virtual and physical encounters were not matched or adjusted for patient characteristics, clinical characteristics, service utilization patterns, or other potential confounders, these findings describe an observed difference in recorded encounter costs rather than a causal cost saving or economic advantage. This is consistent with recent economic evidence showing that telehealth can reduce healthcare costs in selected conditions and delivery models, although the magnitude and certainty of savings vary according to implementation context [20,21]. The largest relative differences in recorded mean cost were observed for Mental Health (66.8%), Neuroscience Centre–Admin (53.6%), and Pediatrics (42.1%). However, the available data did not permit attribution of these differences to specific factors such as staffing, infrastructure, technology, patient travel, or other operational costs. The findings therefore provide a descriptive basis for future economic evaluations incorporating detailed resource use, implementation costs, patient-incurred costs, downstream healthcare utilization, and appropriate adjustment or matching between virtual and physical encounters.

The major strengths of this study are its longitudinal observational evaluation covering November 2021 through July 2025, use of routinely collected real-world institutional data, inclusion of a large tertiary-care virtual nursing program spanning multiple specialties, examination of geographic reach and operational performance, and comparison of virtual and physical encounter costs across 11 service lines. The study also has several limitations, including its retrospective observational design, the incomplete and non-equivalent observation periods across calendar years, variation in the period of operation across individual virtual clinics, incomplete or non-exhaustive demographic fields, reliance on a telephone-based program that may limit generalizability to video-centered models, and limited ability to infer causality from temporal associations. In addition, the cost comparison represents a descriptive comparison of recorded mean encounter costs rather than a matched or adjusted economic evaluation and does not account for potential differences in patient and service characteristics or, where unavailable in the source dataset, fixed, start-up, technology, patient-incurred, and downstream costs. Because the available monthly activity series began in November 2021 and the study period ended in July 2025, the dataset did not represent five complete calendar years. Findings were therefore interpreted according to the observed periods available for each analysis rather than as comparisons of equivalent full-year periods. The longitudinal regression and correlation analyses were exploratory because the number of available clinic-year observations varied across clinical groups and was limited for several groups. The study also had variable completeness of demographic and geographic information across calendar years. Differences in data completeness may affect longitudinal comparisons of patient characteristics and geographic distribution, particularly where the proportion of patients with available information changed over time. The geographic findings therefore describe the distribution of recorded patients with available geographic information rather than standardized changes in the underlying population served. These limitations should be considered when interpreting the observed differences in recorded costs and operational measures and when generalizing the findings to other health systems.

## 5. Conclusions

This evaluation describes the evolution of the Virtual Nursing Program at KFSH&RC from November 2021 to July 2025, including changes in observed patient volume, geographic distribution, consultation modality, clinical-group activity, appointment adherence, and recorded encounter costs. The program showed substantial variation and expansion in recorded activity across clinical groups, accompanied by a broader geographic distribution of patients over the evaluation period. Telephone consultation remained the predominant modality. The cost analysis also showed lower observed mean encounter costs for virtual care than physical care across the evaluated discharge service lines, although these comparisons were not matched or adjusted and therefore do not establish causal cost savings or cost-effectiveness. Further prospective and comparative studies are needed to evaluate clinical outcomes, patient experience, resource utilization, and the full economic consequences of virtual nursing. In particular, future studies should assess whether changes in geographic distribution translate into measurable improvements in access and should evaluate virtual nursing models using a wider range of modalities.

## Figures and Tables

**Table 1:** Yearly demographic characteristics of patients receiving virtual nursing services, November 2021–July 2025.

Year	Unique Patients, n	*Patients Seen, n*	Male, n (%)	Female, n (%)	Missing Sex, n (%)	Saudi, n (%)	Non-Saudi, n (%)	Missing Nationality, n (%)	Median Age, Years	IQR
2021	786	662	295 (37.5)	313 (39.8)	178 (22.7)	333 (42.4)	275 (35.0)	178 (22.6)	37	14
2022	9741	15,323	3828 (39.3)	3747 (38.5)	2166 (22.2)	4831 (49.6)	2744 (28.2)	2166 (22.2)	37	16
2023	6135	32,630	2231 (36.4)	2717 (44.3)	1187 (19.3)	4071 (66.4)	877 (14.3)	1187 (19.3)	38	21
2024	13,792	51,008	5383 (39.0)	7163 (51.9)	1246 (9.1)	11,768 (85.3)	778 (5.6)	1246 (9.0)	40	29
2025	15,672	38,471	6667 (42.5)	8295 (52.9)	710 (4.6)	13,938 (88.9)	1025 (6.5)	709 (4.5)	40	29

Note: The 2021 data represent November–December 2021, while the 2025 data represent January–July 2025; 2022–2024 represent the corresponding full calendar years. Unique Patients represent distinct patients within each calendar year, whereas patients seen represent service activity and may include repeated visits by the same patient.

**Table 2:** Regional distribution of patients receiving virtual nursing services, 2021–2025.

Year	Central Region, n (%)	Northern Region, n (%)	Southern Region, n (%)	Eastern Region, n (%)	Western Region, n (%)	Unknown/Undefined, n (%)	Missing/Unavailable Region
2021	603 (99.2)	—	—	—	2 (0.3)	3 (0.5)	178 (22.6%)
2022	7216 (95.3)	100 (1.3)	102 (1.3)	73 (1.0)	65 (0.9)	19 (0.3)	2166 (22.2%)
2023	3550 (71.7)	451 (9.1)	416 (8.4)	290 (5.9)	219 (4.4)	22 (0.4)	1187 (19.3%)
2024	7475 (59.6)	1491 (11.9)	1528 (12.2)	1161 (9.3)	792 (6.3)	99 (0.8)	1246 (9.0%)
2025	9048 (60.5)	1691 (11.3)	1883 (12.6)	1300 (8.7)	938 (6.3)	103 (0.7)	709 (4.5%)

Note: Percentages for reported regions were calculated among patients with available/codable regional information. The missing/unavailable category is reported separately as a proportion of all unique patients in the corresponding year.

**Table 3:** Comparison of recorded encounter volumes and mean costs between physical and virtual clinics by discharge service line.

Discharge Service Line	Physical Encounters, n	Physical Total Cost	Recorded Mean Cost per Encounter (Saudi Riyals)	Virtual Encounters, n	Virtual Total Cost	Virtual Cost/Visit	Relative Difference in Recorded Mean Cost per Encounter, %
Family Medicine/Polyclinics	73,544	44,489,693	605	19,917	11,543,746	580	4.1
Medicine	71,422	78,930,918	1105	11,513	8,304,840	721	34.8
Obstetrics and Gynecology	59,986	33,226,246	554	5657	2,344,966	415	25.1
Oncology Centre	41,203	42,605,650	1034	2667	2,286,753	857	17.1
Pediatrics	27,339	40,847,641	1494	1944	1,682,470	865	42.1
Urology	13,641	14,293,679	1048	742	593,920	800	23.7
Orthopedic Surgery	12,955	25,196,899	1945	770	1,062,969	1380	29.1
Dermatology	9780	7,087,095	725	333	166,263	499	31.2
Otolaryngology	9573	9,579,035	1001	44	32,079	729	27.2
Neuroscience Centre–Admin	7301	5,218,830	715	2501	829,867	332	53.6
Mental Health	4966	8,500,244	1712	257	146,035	568	66.8
Total	331,710	309,975,929	934.48	46,345	28,993,908	625.61	33.1

## Data Availability

The data that support the findings of this study are available upon request from the corresponding author. The data are not publicly available due to privacy or ethical restrictions.

## Supplementary Materials

The following supporting information can be downloaded at: https://www.mdpi.com/article/10.3390/healthcare14193181/s1, Table S1: Summary of analytical datasets and units of analysis; Table S2: City-level distribution of patients receiving virtual nursing services by year; Table S3: Monthly number of patients receiving virtual nursing services, November 2021–July 2025; Table S4: Virtual nursing appointment volume by clinical group, November 2021–July 2025. Table S5. Median and interquartile ranges (IQRs) of clinic-year patient volume and no-show rates across virtual nursing groups, November 2021–July 2025; Table S6: Longitudinal change in clinic-level virtual nursing activity among comparable baseline and 2025 observations; Table S7: Linear trends in no-show rates by clinical group; Table S8: Spearman correlation between appointment volume and no-show rate by clinical group.

## Abbreviations

The following abbreviations are used in this manuscript:

Abbreviation	Definition
HHC	Home Health Care
IQR	Interquartile Range
KFSH&RC	King Faisal Specialist Hospital & Research Centre
VAD	Ventricular Assist Device
