# Supplementary material for: Evolution of a Large-Scale Virtual Nursing Program: Utilization, Geographic Reach, and Cost Comparison at a Tertiary Care Center in Saudi Arabia, November 2021–July 2025
Source: Healthcare (Basel). 2026 Sep 25;14(19):3181. doi: 10.3390/healthcare14193181 (PMC13665213; doi:10.3390/healthcare14193181)
Supplement: Supplementary file 1 [file healthcare-14-03181-s001.zip › healthcare-4545000-supplementary.pdf]

Table S1. Summary of analytical datasets and units of analysis.

Dataset / analysis	Source / extract	Observation period	Unit of analysis	Principal measure	Denominator
Demographic and geographic	Virtual nursing patient-level extract	November 2021–July 2025	Unique patient within calendar year	Patients, sex, nationality, region, city	Unique patients with available information
Consultation modality	Consultation-type extract	November 2021–July 2025	Consultation	Telephone/video consultation	Recorded consultations in extract
Clinical-group activity	Appointment extract	November 2021–July 2025	Appointment	Appointment volume by clinical group	Recorded appointments in extract
No-show / patient-seen	Appointment-status extract	Available clinic-year observations	Clinic-year / appointment status	No-show and patient-seen rates	Eligible scheduled appointments
Longitudinal association	Clinic-level operational dataset	Group-specific available years	Clinic-year observation	Volume and no-show rate	Available clinic-year observations
Cost comparison	Administrative/financial extract	Available study-period records	Encounter	Cost per virtual/physical encounter	Included encounters by care modality

Note: The analytical datasets represent different units and denominators and were analyzed separately. Multiple appointments, consultations, or encounters may correspond to the same patient. Clinic-year observations represent repeated observations of clinics or clinical groups across available calendar years.

Table S2. City-level distribution of patients receiving virtual nursing services by year.

Year	City	*n*	%
2021	Riyadh	608	99.9
	Medina	1	0.2
2022	Riyadh	7,101	97.3
	Buraydah	28	0.4
	Abha	27	0.4
	Hail	25	0.3
	Sakaka	23	0.3
	Alahsa	20	0.3
	Jeddah	20	0.3
	Dammam	19	0.3
	Arar	18	0.2
	Najran	18	0.2
2023	Riyadh	3,265	80.0
	Hail	120	2.9
	Abha	97	2.4
	Medina	96	2.4
	Buraydah	95	2.3
	Najran	86	2.1
	Jazan	84	2.1
	Sakaka	82	2.0
	Dammam	80	2.0
	Alahsa	77	1.9
2024	Riyadh	6,451	69.6
	Hail	376	4.1
	Buraydah	347	3.7
	Dammam	315	3.4
	Abha	309	3.3
	Medina	303	3.3
	Jazan	300	3.2
	Najran	298	3.2
	Alahsa	297	3.2
	Arar	268	2.9
2025	Riyadh	7,828	70.1
	Buraydah	430	3.9
	Hail	428	3.8
	Abha	416	3.7
	Jazan	378	3.4
	Najran	370	3.3
	Dammam	362	3.2
	Medina	342	3.1
	Alahsa	329	2.9
	Sakaka	279	2.5

Note: City-level percentages use the total number of patients with available and classifiable city information as the denominator for the corresponding year. These denominators may differ from regional-level denominators because the completeness of city and region information was not identical.

Table S3. Monthly number of patients receiving virtual nursing services, November 2021–July 2025.

Year	*Month*	*Patients*
2021	November	169
	December	617
2022	January	878
	February	876
	March	864
	April	725
	May	984
	June	1,214
	July	877
	August	1,688
	September	1,318
	October	1,523
	November	1,890
	December	2,486
2023	January	3,392
	February	2,741
	March	3,047
	April	1,597
	May	3,199
	June	2,486
	July	2,250
	August	2,833
	September	2,558
	October	3,131
	November	2,849
	December	2,547
2024	January	3,459
	February	3,386
	March	2,786
	April	2,839
	May	4,538
	June	2,862
	July	4,801
	August	4,812
	September	4,898
	October	5,899
	November	5,050
	December	5,678
2025	January	5,547
	February	5,007
	March	4,392
	April	5,315
	May	6,222
	June	4,839
	July	7,149

Table S4: Virtual nursing appointment volume by clinical group, November 2021–July 2025.

Clinical group	*No. of clinics*	*2021*	*2022*	*2023*	*2024*	*2025*	*Total*
Cancer Center of Excellence	14	0	1,494	2,237	5,684	2,324	11,739
Medical Nursing	13	980	13,961	5,749	5,964	3,676	30,330
Home Health Care	3	1	1,720	1,125	3,289	972	7,107
Genetics	1	0	4	70	74	50	198
Obstetrics & Gynecology	4	0	0	3,671	6,166	0	9,837
Wound–Stoma Care	2	0	0	9	63	0	72
Neurosciences	1	0	20	105	628	533	1,286
Gastroenterology	2	0	43	140	6,659	0	6,842
ORGAN TRANSPLANT CENTRE OF EXCELLENCE	2	0	0	238	1,027	101	1,366
Surgery–Perioperative	4	0	631	8,122	12,239	59	21,051
Urology	2	0	0	0	594	212	806
Cardiology	3	0	0	8	497	766	1,271
Tracheostomy–Airway Care	1	0	0	0	78	0	78
Pediatrics (General)	6	0	35	232	1,913	528	2,708
Palliative Care	2	0	0	0	594	1,338	1,932
Patient & Family Education	2	0	0	0	537	405	942
Total	**—**	**981**	**17,908**	**21,706**	**46,006**	**10,964**	**87,565**

Note: Appointment counts are aggregated to the 16 major clinical groups and do not represent a count of individual virtual clinics.

Table S5. Median and interquartile ranges (IQRs) of clinic-year patient volume and no-show rates across virtual nursing groups, November 2021-July 2025.

Clinical group	***Patient volume, median [IQR]***	***No-show rate, median [IQR]***
Cancer Center of Excellence	1,845 [1,288–2,055]	15.4% [12.7–17.3]
Medical Nursing	5,579 [3,563–5,754]	3.5% [3.1–7.2]
Home Health Care	1,087 [968–1,560]	1.3% [0.4–3.4]
Genetics	45 [4–66]	6.2% [2.9–8.4]
Obstetrics & Gynecology	0 [0–3,626]	1.7% [1.2–2.1]
Wound–Stoma Care	0 [0–8]	13.5% [11.1–15.9]
Neurosciences	103 [17–476]	8.7% [4.3–12.8]
Gastroenterology	40 [0–139]	5.5% [0.7–7.0]
Organ Transplant Centre of Excellence	91 [0–219]	8.0% [0.9–9.9]
Surgery–Perioperative	606 [53–8,066]	2.5% [0.8–7.1]
Urology	0 [0–207]	5.6% [2.4–8.9]
Cardiology	6 [0–455]	9.1% [8.5–25.0]
Tracheostomy–Airway Care	0 [0–0]	23.1% [23.1–23.1]
Pediatrics (General)	194 [31–497]	10.2% [7.5–13.9]
Palliative Care	0 [0–582]	2.1% [2.0–2.1]
Patient & Family Education	0 [0–358]	9.2% [6.7–11.6]

Table S6. Longitudinal change in clinic-level virtual nursing activity among comparable baseline and 2025 observations.

Clinical group	Baseline year	Baseline activity	2025 activity	Absolute change	% change
Cancer Center of Excellence	2022	1,288*	2,055*	+767	+59.5
Medical Nursing	2021	868	3,563	+2,695	+310.5
Home Health Care	2021	1	968	+967	+96,700
Genetics	2022	4	45	+41	+1,025
Obstetrics & Gynecology	2023	3,626	0	−3,626	−100.0
Wound–Stoma Care	2023	8	0	−8	−100.0
Neurosciences	2022	17	476	+459	+2,700
Gastroenterology	2022	40	0	−40	−100.0
ORGAN TRANSPLANT CENTRE OF EXCELLENCE	2023	219	91	−128	−58.4
Surgery–Perioperative	2022	606	53	−553	−91.3
Urology	2024	334	207	−127†	−38.0†
Cardiology	2023	6	696	+690	+11,500
Tracheostomy–Airway Care	2024	60	0	−60	−100.0
Pediatrics (General)	2022	31	497	+466	+1,503.2
Palliative Care	2024	582	1,310	+728	+125.1
Patient & Family Education	2024	501	358	−143	−28.5

Note: For each clinical group, the baseline year was defined as the first year in which the relevant activity measure was recorded. The longitudinal comparison included groups for which the corresponding activity measure was available at both the baseline year and 2025. Values are derived from the restricted clinic-level longitudinal dataset and are not directly equivalent to the broader clinical-group appointment totals.

Table S7. Linear trends in no-show rates by clinical group.

Clinical group	*Years available*	*Slope/year*	*P value*	R^2
Cancer Center of Excellence	4	−0.01517	0.0027	0.995
Medical Nursing	5	−0.01682	0.178	0.506
Home Health Care	5	−0.00390	0.668	0.069
Genetics	4	+0.03104	0.0381	0.925
Obstetrics & Gynecology	2	+0.03027	—	1.000
Wound–Stoma Care	2	+0.02402	—	1.000
Neurosciences	4	−0.00813	0.812	0.035
Gastroenterology	3	−0.00125	0.958	0.004
ORGAN TRANSPLANT CENTRE OF EXCELLENCE	3	+0.00959	0.855	0.051
Surgery–Perioperative	4	+0.02263	0.281	0.517
Urology	2	−0.04746	—	1.000
Cardiology	3	−0.07009	0.439	0.596
Pediatrics (General)	4	−0.02625	0.304	0.484
Palliative Care	2	+0.05379	—	1.000
Patient & Family Education	2	+0.01868	—	1.000

Note: These analyses are exploratory and hypothesis-generating. P values are unadjusted for multiplicity and should not be interpreted as confirmatory evidence. The number of available annual observations was limited and varied across clinical groups.

Table S8. Spearman correlation between appointment volume and no-show rate by clinical group.

Clinical group	n	Spearman r	P value
Cancer Center of Excellence	37	0.333	0.044
Medical Nursing	24	0.362	0.083
Home Health Care	11	0.706	0.015
Genetics	4	0.400	0.750
Obstetrics & Gynecology	6	0.029	1.000
Wound–Stoma Care	3	0.500	1.000
Neurosciences	4	−0.400	0.750
Gastroenterology	6	0.290	0.577
ORGAN TRANSPLANT CENTRE OF EXCELLENCE	4	−1.000	0.083
Surgery–Perioperative	8	0.268	0.520
Urology	3	0.500	1.000
Cardiology	7	−0.429	0.354
Tracheostomy–Airway Care	1	Not estimated	Not estimated
Pediatrics (General)	12	0.063	0.852
Palliative Care	4	−0.800	0.333
Patient & Family Education	4	0.400	0.750
